# The Spectrum of Design Solutions for Improving the Activity-Selectivity Product of Peptide Antibiotics against Multidrug-Resistant Bacteria and Prostate Cancer PC-3 Cells

**DOI:** 10.3390/molecules25153526

**Published:** 2020-08-01

**Authors:** Davor Juretić, Anja Golemac, Denise E. Strand, Keshi Chung, Nada Ilić, Ivana Goić-Barišić, François-Xavier Pellay

**Affiliations:** 1 Mediterranean Institute for Life Science, 21000 Split, Croatia; anja.golemac@medils.hr (A.G.); denise.strand@gmail.com (D.E.S.); keshi.chung@medils.hr (K.C.); fxpellay@medils.hr (F.-X.P.); 2 Department of Physics, Faculty of Science, University of Split, 21000 Split, Croatia; nada.ilic.pekic@gmail.com; 3 Department of Clinical Microbiology, Split University Hospital and Split University Medical School, 21000 Split, Croatia; ivanagoicbar@net.hr; 4 Naos Institute of Life Science, 13593 Aix-en-Provence, France

**Keywords:** antimicrobial peptides, anticancer peptides, therapeutic index, design solutions, selectivity

## Abstract

The link between the antimicrobial and anticancer activity of peptides has long been studied, and the number of peptides identified with both activities has recently increased considerably. In this work, we hypothesized that designed peptides with a wide spectrum of selective antimicrobial activity will also have anticancer activity, and tested this hypothesis with newly designed peptides. The spectrum of peptides, used as partial or full design templates, ranged from cell-penetrating peptides and putative bacteriocin to those from the simplest animals (placozoans) and the Chordata phylum (anurans). We applied custom computational tools to predict amino acid substitutions, conferring the increased product of bacteriostatic activity and selectivity. Experiments confirmed that better overall performance was achieved with respect to that of initial templates. Nine of our synthesized helical peptides had excellent bactericidal activity against both standard and multidrug-resistant bacteria. These peptides were then compared to a known anticancer peptide polybia-MP1, for their ability to kill prostate cancer cells and dermal primary fibroblasts. The therapeutic index was higher for seven of our peptides, and anticancer activity stronger for all of them. In conclusion, the peptides that we designed for selective antimicrobial activity also have promising potential for anticancer applications.

## 1. Introduction

Antimicrobial peptides (AMPs) are small (5–50 amino acid residues), mostly cationic and amphipathic molecules, often associated with a broad activity spectrum against different cell types [1,2,3,4]. As host defense peptides (HDPs), they occur naturally as part of the innate immune defense, for instance, in multicellular organisms [5]. Such peptides are easy to synthesize, with a general mechanism of membrane-perturbing activity. Cationic AMPs attach themselves to negatively charged bacterial membranes. When critical surface concentration is reached, a range of different perturbing activities can occur, including short-lived pore formation, leading to the quick inhibition of bioenergetics, almost instant cessation of growth, and a radical decrease in the number of living cells [1,2,6,7]. The main design challenge in this research field is to simultaneously achieve the goals of wide-spectrum antimicrobial activity and low toxicity to human cells. For possible medical applications, activity against multidrug-resistant bacteria is also a highly worthwhile goal.

In addition to their antimicrobial action, other applications have been discovered for both natural and designed AMPs. These include anti-inflammatory, antitoxic, antioxidant, wound healing, antiviral, antifungal, antiparasitic, and anticancer properties [2,8]. Almost as soon as the first AMPs were discovered in amphibians, it became clear that their membrane-poration activity extends to normal and cancerous human cells [9,10]. Such a general membrane-disturbing activity mechanism raised hopes of defeating the resistance pathways connected to mutations in specific protein targets induced by other antibiotic and anticancer drugs [11].

In this work, we examined whether highly active and highly selective antibiotic peptides are also active and selective against cancer cells. We set out to investigate if the similarly potent antibacterial activity of designed highly cationic but non-homologous helical peptides is the predictor of their anticancer activity or selectivity. On the APD3 (antimicrobial peptide database [12]), 230 antimicrobial peptides are currently listed as anticancer peptides (ACPs) at the time of writing. Among anuran HDPs, at least 108 have anticancer activity (DADP database [13]). The DBAASP database of natural and artificial AMPs contains 1406 peptides, with dual antimicrobial and anticancer activity [14].

Cancer is the major leading cause of death in the population under 70 in over 90 countries, and is expected to become the greatest obstacle to increasing longevity in the 21st century [15]. Both incidence and mortality are on the rise worldwide. Current treatments often lack strong cancer selectivity, giving rise to side effects by damaging healthy tissue. Some treatments are also rendered ineffective by the development of resistance over time [1,2,3,8,16,17]. As such, new therapeutic approaches with alternative mechanisms are necessary and under development [8,17].

As the mechanisms of action differ from that of conventional chemotherapy, AMPs offer a potential alternative [8]. Some AMPs target cancer cells using the same mechanism by which they act as bactericides. The net negative charge, increased surface area, and fluidity of the membrane of cancer cells differs from that of healthy cells, and makes them more attractive and susceptible to the peptides [1,2,3,8,18]. Since AMPs act as receptor-independent membrane permeators, there is a low risk of development of resistance. Multidrug resistance is most commonly caused by the expression of efflux pumps that expel detected anticancer drugs from the cell. This mechanism does not apply to ACPs, as they target the membrane and thus destabilize cell integrity [19]. This mechanism of action is also the reason for their broad specificity toward different targets [7,8,17,20,21]. ACPs could be included in a potential combined therapy, to be used in tandem with conventional chemotherapy by compromising membrane integrity, which would allow other anticancer agents to enter tumor cells with more ease at lower concentrations [7,21].

This paper aims to present the design and multifunctional screening of nine AMPs, some new and some described in our recent publications [22,23,24], for their in vitro performance as wide-spectrum antimicrobial and anticancer peptides. We define the overall performance as the product of peptide activity and selectivity. It is examined firstly in a variety of prokaryotic cells, including Gram-negative, Gram-positive, and multidrug-resistant bacteria. The design approach takes advantage of evolutionary mechanisms in creating widely distributed defense peptides found in almost all species, including AMPs from bacteria (bacteriocins), from the most primitive animals, and from the Chordata phylum (anurans) richly endowed with HDPs. We describe how natural or designed peptides are used as templates to introduce amino acid substitutions predicted by custom designer tools, to attain better overall antimicrobial performance. We reasoned that an excellent wide-spectrum performance against bacteria with net negative surface charge is likely to indicate activity against cancer cells, and we compared our nine designed peptides with a known ACP to define their anticancer activity.

## 2. Materials and Methods

### 2.1. Chemicals (Reagents)

RPMI-1640 Medium (R0883), Dulbecco’s Modified Eagle’s Medium (DMEM), Dulbecco’s Phosphate Buffered Saline (PBS) and dimethyl sulfoxide (DMSO) were obtained from Sigma-Aldrich. Fetal bovine serum, L-Glutamine (200 mM), Penicillin-Streptomycin and Trypsin-EDTA (0.25%, with phenol red) were obtained from Gibco by Life Technologies. Thiazolyl Blue Tetrazolium Bromide used in cytotoxicity assays and Triton™ X-100 used in hemolysis assays were obtained from Sigma-Aldrich. EDTA powder (>99% pure) was obtained from Bio-Rad. Tryptone (211705, Bacto^TM^) and yeast extract (212750, Bacto^TM^) were purchased from BD Biosciences. Peptides were obtained from GenicBio Limited (Shanghai, China).

### 2.2. Peptide Design Methods

Primary structures of designed peptides are listed in Table 1, together with highlighted substitutions or additions with respect to parent peptides. Parent peptides were either natural or designed antimicrobial peptides. The detailed design method is described in the results part. Briefly, we started with naturally evolved known or suspected host defense peptides, and used a combination of expert knowledge and home-developed algorithms to increase the activity and selectivity of peptide analogs regarding considered parent peptides. Increasing net charge, hydrophobicity, hydrophobic moment, or predicted selectivity were design approaches used alone or combined with the goal to increase selectivity for anionic membranes. Note that the selectivity index abbreviation (SI) is used together with the therapeutic index abbreviation (TI), which appears in some of the cited papers, but the mathematical meaning is identical in our context: the ratio of peptide concentration toxic to healthy human cells to peptide concentration toxic to bacteria or cancer cells. The choice of healthy human cells differed when antimicrobial and anticancer selectivity was examined. Erythrocytes served for the SI calculation regarding bacteria, while primary dermal fibroblasts were used to calculate TI for cancer cells.

We used our freely available online software tools to select for and to check the progress after each design step:

Mutator tool [25] server: http://mutator.djpept.com/ or http://splitbioinf.pmfst.hr/mutator/.

Therapeutic Index Estimator [26] server: http://splitbioinf.pmfst.hr/split/dserv1/.

MIC-predictor server [27]: http://micpredictor.djpept.com/ or http://splitbioinf.pmfst.hr/micpredictor/.

DADP database of anuran defense peptides [13]: http://split4.pmfst.hr/dadp/.

SPLIT server 3.5 [28] for the predicting sequence profile of hydrophobicities, optimal hydrophobic moments, and membrane preference for amphipathic and membrane-associated helical segments (the default choice for all profiles): http://split.djpept.com/split/ or http://splitbioinf.pmfst.hr/split/.

SPLIT server 4.0, which performs an automatic selection of optimal amino acid attribute (hydrophobicity scale) and corresponding preference functions, to predict the sequence location of the membrane-buried helical segments and transmembrane topology of integral membrane proteins [29] http://split.djpept.com/split/4/ or http://splitbioinf.pmfst.hr/split/4/.

The following online bioinformatic tools were used for predicting:

(a) physicochemical properties of helical peptides based on their sequence:
  HeliQuest tool [30]:
  https://heliquest.ipmc.cnrs.fr/
(b) the probability for a peptide to be an antimicrobial peptide:
  CAMP_R3_ artificial intelligence algorithms for predicting AMPs [31]:
  http://www.camp.bicnirrh.res.in/predict/
(c) the probability for a peptide to be a cell-penetrating peptide:
  Cell-penetrating peptide (CPP) prediction, according to CPPrex-FL [32] and MLCPP [33] algorithms with respective links http://server.malab.cn/SkipCPP-Pred/Index.html and http://thegleelab.org/MLCPP/MLCPP.html.
(d) the probability for a peptide to be an anticancer peptide:
  Anticancer probability servers used were that of:
  http://crdd.osdd.net/raghava/anticp/ [34],
  http://codes.bio/acpred/ [35],
  www.thegleelab.org/mACPpred [36].

### 2.3. Bacterial Strains and Antimicrobial Activity Assay

The American Type Culture Collection (ATCC, Rockville, MD, USA) strains and *Escherichia coli* MG1655 were used as standard bacterial strains. Gram-negative ATCC strains consisted of *Escherichia coli* (ATCC 25922), *Pseudomonas aeruginosa* (ATCC 27853), *Acinetobacter baumannii* (ATCC 19606), and *Klebsiella pneumoniae* (ATCC 13883). The only Gram-positive standard strain chosen for testing was *Staphylococcus aureus* (ATCC 29213). Clinical isolates with confirmed multidrug-resistance phenotype were obtained from different wards of the University Hospital Center, Split, Croatia. We described their origin, antibiograms, and resistance phenotype previously [24]. In the same paper, we described the procedure for antimicrobial susceptibility testing on planktonic cells, using the microdilution method in 96-well microtiter plates.

Briefly, cells were grown on agar plates and a single colony was sampled to generate a liquid culture (LC) grown overnight at 37 °C. The culture was sampled, resuspended in fresh medium, and allowed to grow at 37 °C, until reaching the mid-exponential phase. Lysogeny broth (LB) was used in the case of the *E. coli* MG1655 cells. Mueller Hinton (MH) medium was used for all other bacterial strains. Serial 2-fold dilutions of the peptide (64 μM to 0.0625 μM) were prepared from peptide stock solution in PBS, and added to a final load of 5 × 10^5^ CFU/mL in 100 μL total volume of medium per well in a 96-well plate (Brand 781660). Bacterial cultures were incubated with peptides for 18 h to 24 h at 37 °C. The minimum inhibitory concentration (MIC) was defined as the lowest concentration of the peptide, at which no visible bacterial growth was observed. The MIC for each of the tested peptides was determined in at least three individual assays under the same laboratory conditions. For the determination of minimum bactericidal **c**oncentration (MBC), 4 μL aliquots were taken from the wells corresponding to MIC, 2 × MIC, and 4 × MIC, and then plated on MH agar plates. After incubation for 18 h at 37 °C, the MBC values were recorded as concentrations, causing a ≥99.9% killing of the initial inoculum, which contained about 2000 CFU. At the end of the incubation period for MIC or MBC, the determination of the OD600 was analyzed.

### 2.4. Cytotoxicity on Cancer Cells and Fibroblasts

Prostate cancer cells were donated by the University of Split, School of Medicine. Cells were cultured in RMPI-1640 medium, supplemented with 10% FBS, 1% Penicillin-Streptomycin, and 1% L-Glutamine. Human dermal fibroblasts were purchased from Biopredic (France) and cultured in DMEM medium, supplemented with 10% FBS and 1% Penicillin-Streptomycin. All cell lines were maintained in a humidified incubator with 5% CO_2_ at 37 °C. Cells were detached using the trypsin-EDTA solution and sub-cultured (7000 cells/well) in a 96-well plate overnight. Peptide stock solutions were prepared in PBS, and cancer cells were treated with 2-fold serial dilutions for 24 h at 37 °C, with 5% CO_2_. The toxicity of 6 selected peptides was assessed by a standard MTT assay. Treated cells were incubated with MTT working solution (0.5 mg/mL Thiazolyl Blue Tetrazolium Bromide) for 4 h at 37 °C with 5% CO_2_. Precipitated formazan was dissolved in 200 µL DMSO and absorbance was measured at 595 nm with EnSight Multimode Plate Reader (PerkinElmer, Inc.)

### 2.5. Hemolysis of Human Erythrocytes

Fresh blood was obtained from a healthy female donor. Blood was stored in a tube containing EDTA at 4 °C and used within 24 h. Before use, 1 mL of blood was centrifuged (400× *g*, 10 min), and the supernatant was discarded. Moreover, 500 μL cold PBS with EDTA (1 mM) was added, mixed and centrifuged. The supernatant was discarded. Furthermore, 500 μL of cold PBS with EDTA (1 mM) was added, from which a dilution of 1% blood was made. Peptides were prepared in PBS at a 2 × concentration in 100 µL. Then, 100 µL diluted 1% blood was added to the peptide solution for a final RBC concentration of 0.5% of starting concentration. The suspension was incubated at 37 °C for 1 h and centrifuged at 10,000× *g* for 5 min. A parallel incubation with 0.2% Triton was performed to determine the absorbance value associated with 100% hemolysis, and with PBS alone to determine 0% hemolysis. After centrifugation, the supernatant was added to a 96-well microtiter plate (150–180 μL), and the absorbance was measured at 450 nm, using EnSight Multimode Plate Reader (PerkinElmer, Inc.). Each measurement was done in triplicate.

**Figure 1:**
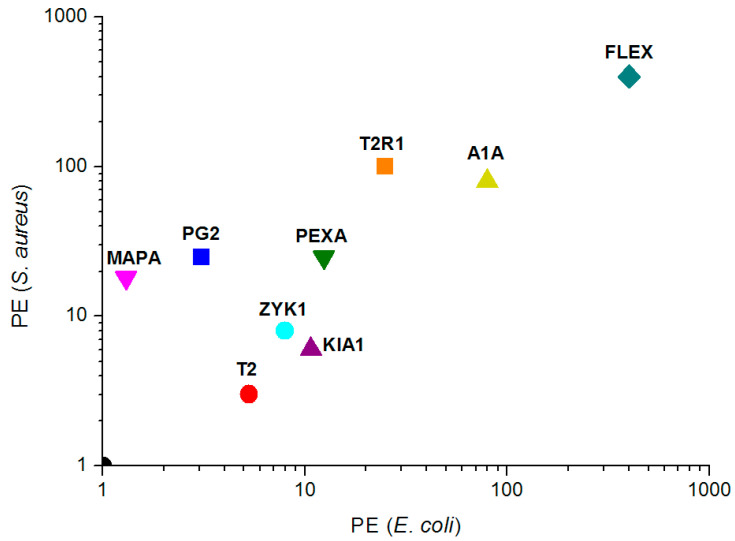
The performance PE (20) of designed peptides (the product of selectivity and antibacterial activity) against standard GRAM- and GRAM+ strains. See Table 1 for peptide abbreviations and Table 2 for performance calculations.

**Figure 2:**
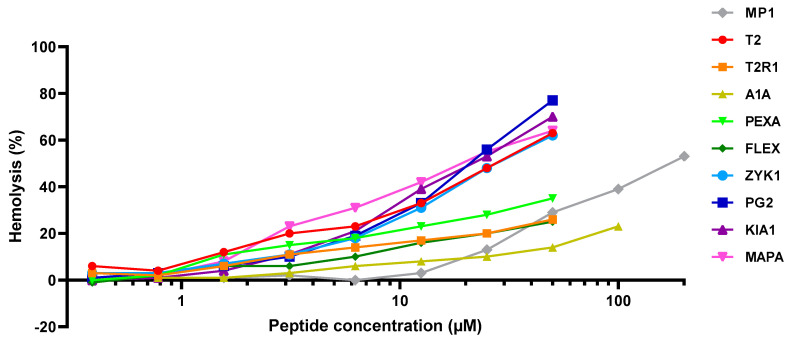
Plots of the hemolytic effect of peptides T2, T2R1, A1A, PEXA, FLEX, ZYK1, PG2, KIA1, and MAPA, compared to MP1 (control) on erythrocytes.

To calculate selectivity or therapeutic index, authors have used different procedures and definitions for unitless numbers, in a whole range from 100% [37], to minimal undetected hemolysis concentration [38], divided by the antibiotic activity. The most common measurement of HC_50_ would require unreasonably large amounts of those peptides that show a weak hemolytic activity, and so HC_5_, HC_10_, and HC_20_ have become preferred measurements to get the first impressions about the selectivity of compounds during recent years [39,40,41,42,43]. We opted to also include the extrapolated HC_50_ in our results, to permit comparisons with older toxicity results. However, the HC_20_ measurements were used for ranking the peptides according to their selectivity and overall performance (Figure 1 and Figure 2 and Table 2).

## 3. Results

### 3.1. Peptide Design

Different design strategies were used, with the main goal of obtaining broad-spectrum peptide antibiotics with minimal inhibitory concentration (MIC) in the micromolar range. To test the design achievements, we used a simple method for estimating the overall antibacterial performance of a considered peptide. Low MIC concentration means high antibacterial activity. Hence, the 1/MIC value represents a peptide activity. High toxicity to human cells should not be the outcome of a design procedure, even for highly active peptides. The hemolysis of healthy human erythrocytes is usually used for quick toxicity tests. A peptide is considered to be non-toxic when red blood cells must be incubated with a high peptide concentration, causing 20% or 50% hemoglobin release (HC_20_ or HC_50_). The selectivity index is calculated as SI (20) = HC_20_/MIC or SI (50) = HC_50_/MIC. Antimicrobial performance is then defined as the product of activity and selectivity: PE = SI/MIC [44]. Nine helical peptides that we found or designed (Table 1) have wide-spectrum antimicrobial performance against standard bacterial strains (Table 2). Additionally, most have a strong bactericidal activity (low MBC values) (not shown). Furthermore, most of them have a primary structure that is non-homologous to known antimicrobial peptides. Structures and activities are presented in this paper for the first time for six of these peptides. We addressed whether some of the best performing peptides (against bacteria) are also active and selective against cancer cells.

We describe here detailed procedures of how two trichoplaxins-2 and the four other novel peptides were constructed in silico in preparation for presenting their anticancer activity. The SMIC composite antimicrobial index is introduced as an additional quantitative parameter for selecting amino acid substitutions, deletions, and insertions (Table 1). It multiplies an approximate alpha-amphipathic moment area from the SPLIT 3.5 INDA profile, with the estimated selectivity index, as calculated by the MUTATOR or TI-estimator and divides the result with predicted minimal inhibitory concentration. Since our MIC-predictor is limited to Rana-box containing peptides, we added the CKITGC Rana-box sequence to all designed peptides, not only to Ranatuerin-2CSa with deleted CKITGC, which served as the template for flexampin.

Trichoplaxin-2 (T2). *Trichoplax adhaerens* from the phylum Placozoa belongs to the simplest and oldest living animals [46,47]. This fragile and barely visible thin sheet of cells is likely to possess a powerful armament of host defense molecules, which is mostly unexplored. One exception is our finding of the trichoplaxin AMP (now renamed as trichoplaxin-1) in the EST database belonging to the *T. adhaerens* [48]. For this work, we examined some translated open reading frames from the *T. adhaerens* genome [46] code for typical tripartite structure: signal peptide, acidic propeptide, and mature antimicrobial peptide, as was found for AMP precursors of anuran and many other AMPs [13,49,50]. One such peptide we found had putative AMP at its C-terminal that we named the trichoplaxin-2. Its sequence is non-homologous to trichoplaxin-1 [48]. The design of trichoplaxin-2 and its analog trichoplaxin-2a consisted of deciding where to cut the peptide N-terminal out of the longer sequence, containing predicted antimicrobial segments. The precursor candidate peptide from the gb|GR418172.1| EST entry in the Library of *Trichoplax adhaerens* cDNA, mRNA sequences is:


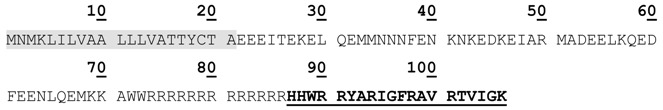


It has a strongly predicted [51] signal peptide (gray shaded N-terminal segment) and well predicted potential AMP [31,52], with a high hydrophobic moment at the C-terminal (bold and underlined). The cut-off between 13 consecutive Arg residues and two His residues was a subjective choice, with no implication that residues 71–86 are less important for antimicrobial or other biological activity.

Trichoplaxin-2A (T2R1) can be considered as a T2 analog with added Arg at its N-terminal, or as another choice of natural peptide obtained with such a cut-off for the precursor N-terminal, that includes the last Arg from the segment with 13 consecutive Arg residues. T2R1 and T2 do not have homology to any other known antibacterial or anticancer peptides.

Adepantin-1 analog (A1A) design had a goal to broaden the activity spectrum of ab initio constructed adepantin-1 peptide GIGKHVGKALKGLKGLLKGLGES [26,53], which was active only against Gram-negative bacteria. Substitutions G3K, H5A, and G19A converted it into A1A: GI**K**K**A**VGKALKGLKGLLK**A**LGES. We have selected lysine and alanine substitutions at sequence positions contributing to predicted selectivity increase [25] and hydrophobic moment increase. Replacing glycine with alanine can increase AMP antimicrobial activity [54]. Except for the adepantin-1 parent peptide, A1A is not similar to any other known peptides. The BLASTP tool found only proteins with E-value equal or higher than 5.5 with the A1A sequence entry.

Pexiganan-L18 (PEXA) sequence GIGKFLKKAKKFGKAFV**L**ILKK was found to increase the predicted selectivity index of the parent pexiganan sequence after Lys-18 to Leu-18 substitution, according to our Therapeutic Index Estimator and Mutator tools [23,25,26]. The TI server [26] predicted SI = 94.90, while the SPLIT 3.5 server [28] predicted increased hydrophobicity and increased helical preference at the peptide C-terminal part. The parent sequence GIGKFLKKAKKFGKAFVKILKK for the well-known peptide antibiotic pexiganan [55,56] has predicted SI = 41.80. Measured MIC = 6.5 μM for standard ATCC *E. coli* and *S. aureus* strains [57] and hemolytic activity of HC_50_ = 45 µM [58] results in the selectivity index SI(50) = 9, and a moderate PE(50) close to 1 (μM)^−1^. Interestingly, none of the three different substitutions offered by the Mutator tool server [21] increased predicted SI to more than SI = 73. This may be due to the known tendency of the algorithm to preferentially introduce substitutions near the peptide N-terminal. Such pexiganan analogs, some of which we tested, had significantly weaker antimicrobial activity undermining any achieved decrease in hemolicity (not shown). Hence, we explored computer-assisted substitutions near the C-terminal of pexiganan. When substitutions for the 18th position only are examined, the K18I and K18V substitutions are predicted to achieve an almost equal selectivity index compared to the K18L substitution (SI = 94.8). All other substitutions in the 18th position produced significantly lower predicted SI. These were rejected, as the main aim of design improvement for pexiganan was to lower its toxicity without decreasing activity.

Zyk-1 (ZYK1) sequence GIGREIIKKIIKKIGKKIGRII started from an already known artificial AMP template, with the sequence GIIKKIIKKIIKKI-NH_2_ [59]. This peptide has weak antimicrobial and anticancer activity. In this case, our design goal was to strengthen both activities by extending its length enough in an assumed helical conformation, to span the hydrophobic membrane barrier easily. Ascaphin N-terminal GFRD was used to elongate the N-terminal of the template peptide with the ascaphin-like GIGRE motif. The reason for replacing D with E is due to the ability of the glutamine residue to decrease the toxicity of AMPs to human cells [60]. Ascaphin is still one of the best natural anuran AMPs [26], although it originated from an ancient family of frogs (*Ascaphidae*), probably more than 100 million years ago [61,62]. Its N-terminal part is more important for membrane-perturbing activity [63]. In the second design step, we used the BLASTP tool to find similar sequences to GIGREIIKKIIKKIIKKI. By far, the best hit (E value = 1 × 10^−6^) was from the hypothetical protein from the ZYK strain of *Bacillus oryziterrae*, with the accession number WP_026092555 [64]. This 60 AA-long peptide sequence is predicted as an AMP with the highest possible score by the Support Vector Machine classifier from the CAMP_R3_ server [31]. Its segment REIIKKIIKKIIKKITKKITKKITKKITKKITREIIREII has an unusually long stretch of 35 highly alpha amphipathic residues, according to the SPLIT 3.5 predictor [28]. The REII motif appears three times, and the [R,K]-XXX-[R,K] motif appears nine times in that peptide, presumably endowing it with some of the numerous biological activities common to helical peptides, with a high hydrophobic moment [65,66]. The modified N-terminal already contains the REII motif, and we subsequently added the shorter RII version to the C-terminal. Additional elongation and predicted higher flexibility was achieved by replacing the Ile with Gly residue at sequence position 15, and by inserting Gly 19 as the last design step, which increased the number of [R,K]-XXX-[R,K] motifs to a total of four. The insertion of glycines 15 and 19 created a small motif G-XXX-G [67], that promotes self-association and dimerization of helical segments in a membrane environment [68]. The resulting sequence **GIGRE**IIKKIIKKI**G**KKI**GRII** had a high predicted selectivity index (SI = 93.7) [25], and a high predicted antimicrobial activity (MIC = 3.2 µM when CKITGQC is added at its C-terminal to enable the prediction of MIC by the MIC-predictor server) [27]. This peptide is rich in G, I, and K amino acid residues, which appear significantly more frequently in anticancer peptides then in peptides devoid of anticancer activity [69].

DiPGLa-H (PG2) and kiadin-1 (KIA1) sequences were published, together with their antimicrobial activity and selectivity testing results [24]. The template for both peptides was an almost inactive PGLa-H sequence KIAKVALKAL from *Xenopus laevis*, which was activated after we designed and tested the sequence tandem peptide, with enough length in helical conformation to span the membrane.

Mapegin (MAPA) design started from the MAP sequence for the cell-penetrating peptide KLALKLALKALKAALKLA [70]. There is some confusion in the literature about the primary structure of the MAP CPP [71]. The presented template sequence is identical to the one published in [72,73]. The design goal was to convert this CPP template into a wide-spectrum but selective antibacterial peptide. HeliQuest, SPLIT, and MUTATOR server results suggested that the template sequence has excessive hydrophobicity and less than optimal selectivity for a peptide antibiotic. The maximal hydrophobicity and the minimal hydrophobic moment values were found near the peptide N-terminal. The omission of Leu-6, and substitutions Leu-2-Ile, Ala-3-Gly, Leu-4-Lys, and Ala-7 to Ile-6 produced the sequence K**IGK**K**I**LKALKAALKLA, with a significantly increased hydrophobic moment, decreased hydrophobicity, and maximal predicted selectivity index. As we intended, the CPP probability decreased for this design intermediate from 1.0 to 0.993, according to the CPP Skip prediction algorithm [27], and from 0.998 to 0.951 according to the CPP MLCPP algorithm [28]. However, AMP prediction [26] did not improve after N-terminal changes in the template sequence, while servers for predicting anticancer peptides produced contradictory results. ACP probability increased from 0.98 to 1.0 [35], but it decreased from 0.85 to 0.74 [34], or from 0.986 to 0.981 [36]. Hence, we introduced the C-terminal changes by using the same method of increasing alpha amphipathic hydrophobic moment, decreasing hydrophobicity, increasing AMP and ACP probability, and decreasing CPP probability. The presence of a Glu residue close to the peptide terminal and increased flexibility was also considered to be beneficial for reaching the stated goal. All these conditions, together with the condition of unchanged peptide length, narrowed the deletions and substitutions choice to the substitution of Ala-13 with Gly-12 and insertion of Glu-16, that produced the final mapegin (MAPA) sequence: K**IGK**K**I**LKALK**G**ALK**E**LA. All four CAMP_R3_ artificial intelligence algorithms [26] now predicted that MAPA would be an antimicrobial peptide. The CPP prediction confidence significantly decreased in accordance with the CPP-AMP transformation goal, from 1.0 to 0.95 in the case of the SkipCPP-Pred algorithm [27], and from 1.0 to 0.7 for the MLCPP algorithm [28]. The Schaduangrat et al. (2019) [35] and Boopathi et al. (2019) [36] servers predicted increased ACP probability with respect to the MAP template, also in accordance with the design goal. There was a significant increase in the alpha amphipathic hydrophobic moment (SPLIT and HeliQuest results), while the estimated selectivity index of SI = 83 left the possibility for the Mutator algorithm to predict additional selectivity, increasing substitutions. All such suggestions would destroy the single remaining small motif AXXXA. Hence, we stopped with substitutions at this point, because of the possible importance of that motif for peptide dimerization in the membrane environment. The SMIC parameter increased from 92 for the MAP peptide to 108 after N-terminal changes, and to 253 after C-terminal changes when MAPA was constructed. This parameter also increased for all other designed peptides, except for T2R1—the peptide which is not anuran-like. MAPA has limited similarity to several maximins (E-value greater than 0.08), antimicrobial peptides from the toad *Bombina maxima*.

Published observations together with theoretical tools (Table 1 [74]) suggest that membrane-associated conformation is helical for all of our nine peptides, despite a high probability of random coil conformation in water solution. CD and NMR spectra [24,75] confirmed that at least 75% of the *Di*PGLa-H and kiadin-1 sequences adopt a helical conformation in membrane-mimicking solvent trifluoroethanol (TFE) or SDS micelles, in accordance with the SPLIT tool prediction for the membrane-buried amphipathic helix length of 75 to 80% (Table 1). MD simulations also predict the 70 to 75% α-helical conformation for these two peptides in TFE and the hydrophobic core of the phosphatidylcholine membrane [24]. Fitting the CD spectra for Polybia-MP1 in anionic vesicles suggested a helical content of 47% or higher [76]. In 50% TFE, the helix content was 58% [77]. These observations can be compared with predicted amphipathic helical length of 50% for that peptide associated with a membrane. When MD simulations were performed for flexampin attached to or inserted into the anionic 1-palmitoyl-2-oleoyl-sn-glycero-3-phosphoethanolamine:1-palmitoyl-2-oleoyl-sn-glycero-3-phosphoglycerol membrane, up to 85% of α-helical conformation was observed [22], well in concord with the 82% prediction by SPLIT. Accordingly, helical conformation for the remaining six peptides when attached to an anionic membrane (Table 1) is a reasonable assumption.

### 3.2. The Performance Parameters for Ranking Peptides When Antibacterial Activity and Toxicity to Human Erythrocytes are Both Taken into Account

The MIC and MBC concentrations were presented in our earlier publications for kiadins [24] and flexampin [22]. In this paper, we show that two trichoplaxin-2 peptides (T2 and T2R1), a designed adepantin-1 analog (A1A), pexiganan analog (PEXA), MAP analog (MAPA), and ZYK1 have equally good activity in a low micromolar range from 0.25 to 2 μM (Table 2). In general, they were only slightly less active against different multidrug-resistant species, which we previously characterized [22,24]. For the selectivity estimate, MIC concentrations for nine helical peptides (Table 1) are compared to concentrations, causing 10%, 20%, and 50% hemolysis of human erythrocytes (Table 2).

The antimicrobial performance ranking for the nine peptides is presented in the PE_a_/PE_c_ diagram (Figure 1). A convenient reference for ranking peptides is to determine whether their performance is better than one of the most promising broad-spectrum AMPs for clinical applications, the MSI-78 peptide (commercially known as pexiganan). All nine of our peptides perform better in vitro than pexiganan. The PE (20) parameters cannot rank pexiganan higher from the point of origin in Figure 1. The distance from that point ranks peptides between five (T2) and more than five hundred times (FLEX), relative to the performance of pexiganan. The best are ranked as FLEX > A1A > T2R1 > PEXA (Figure 1). However, if a different standard strain (*E. coli* MG1655) is used for selectivity and performance calculations, FLEX would not be ranked as the best peptide. For the goal of this paper, the interesting point is that the best peptides (including ZYK1) have almost equally good performance against Gram-negatives and Gram-positives, with a possible preference toward membranes with a higher percentage of anionic polar lipids, such as found in Gram-positive strains [42]. A caveat to take into account here is that the therapeutic index can vary as much as 4-fold when MHC and MIC measurements are carried out by serial 2-fold dilutions [38]. Variations are augmented during performance calculations due to quadratic MIC dependence. Only the MAPA peptide exhibited a clear performance gain (36-fold factor) when tested on a Gram-positive strain.

The high estimated HC_50_ values of T2R1, A1A, FLEX, and PEXA (Table 2) indicates the low toxicity of these peptides. Together with low corresponding MIC values, this observation confirms that significant improvement has been achieved during the design procedure. Trichoplaxin-2 (T2) is much more toxic to erythrocytes than T2R1. The addition of Arg residue to the N-terminal of T2 makes the T2R1 peptide at least one order of magnitude more specific in its antimicrobial activity, without any activity decrease. Adepantin-1 is less active than A1A against *E. coli* strains and almost completely inactive against Gram-positives [26]. As we intended, the specificity for Gram-negatives is lost for its A1A analogue, while high antibacterial selectivity and low cytotoxicity are retained. The gains in FLEX performance with regard to its template peptide, rantuerin-2CSa, have been discussed in our previous publications [44,74]. Performance of PEXA is at least 20-times better from pexiganan, due to more potent activity and higher selectivity.

None of the tested multidrug-resistant clinical isolates were able to grow in the presence of at least one of four peptides T2R1, A1A, FLEX, and PEXA, applied in low micromolar concentration (Table 2). A decades-long study of pexiganan established its effectiveness against over three thousand clinical isolates, including multiresistant strains [56]. Thus, the peptides presented here can also be subjected to further testing regarding potential clinical applications. In this work, we chose to test their anticancer activity and selectivity.

To summarize, we observed that the overall antibiotic activity of designed peptides is high, while toxicity on hematocytes is mostly low (Figure 2 and Table 2). The antibacterial selectivity index is found to cover a wide range. Notably, all of the newly designed peptides are non-homologous to previously known antimicrobial or anticancer peptides, but all of them are predicted to have an additional anticancer activity (Table 1).

### 3.3. Activity and Selectivity against Prostate Cancer Cells

An example of an AMP with subsequently proven anticancer activity in addition to antibacterial activity is the Polybia-MP1 peptide (Table 1 last row). MP1 was previously isolated [45] from among other multifunctional host defense peptides from the venom of the social wasp *Polybia paulista*. The antimicrobial activity of Polybia-MP1 is very diverse. The MIC values range from 3 to 50 μM against Gram-positive and Gram-negative bacteria [78,79], fungi [80], and *Trypanosoma cruzi* parasite [81]. The important additional advantage of low hemolytic activity [77,78] is in accord with our results (Table 2). MP1 presents in vitro selective toxicity to prostate, bladder, and leukemic cancer cells [82,83,84], and in vivo anticancer activity [77]. Therefore, MP1 is a suitable choice for an AMP with a well confirmed selective anticancer activity that can be used as a control when compared to the anticancer performance of our nine peptides.

**Figure 3:**
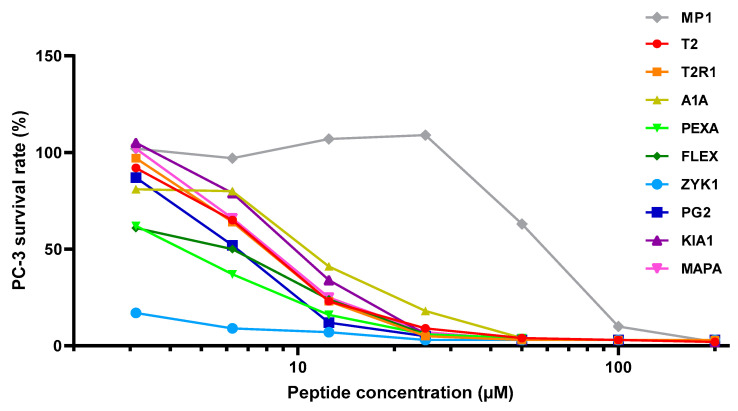
Plots of the activities of peptides T2, T2R1, A1A, PEXA, FLEX, ZYK1, PG2, KIA1, and MAPA compared to MP1 (control) on PC-3 prostate cancer cell line.

**Figure 4:**
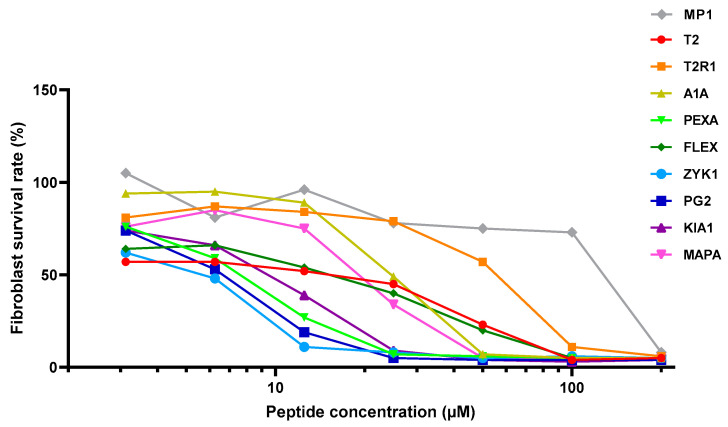
Plots of the activities of peptides T2, T2R1, A1A, PEXA, FLEX, ZYK1, PG2, KIA1, and MAPA compared to MP1 (control) on human primary dermal fibroblasts.

**Figure 5:**
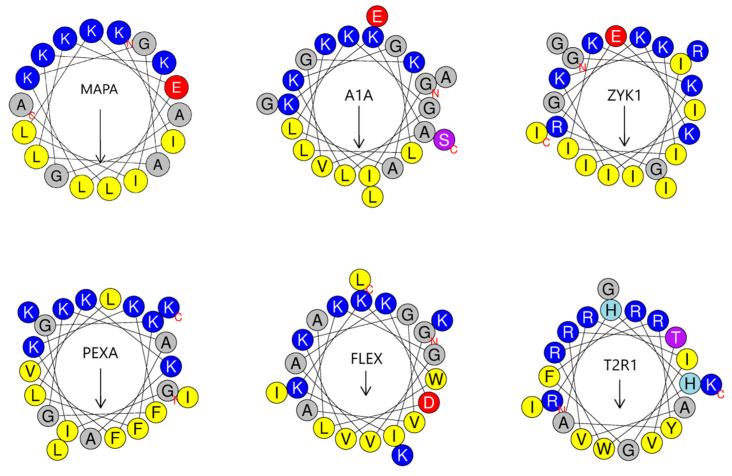
The Schiffer−Edmundson wheel projection of the six best anticancer peptide antibiotics: ZYK1, A1A, MAPA, T2R1, FLEX, and PEXA, drawn by using the HeliQuest tool. See Table 1 for peptide abbreviations. Color code follows the HeliQuest convention: yellow for hydrophobic, blue for cationic, red for anionic, grey for small amino acids, purple for uncharged polar residues, and light blue for histidine. The arrow length and direction indicate that the mean hydrophobic moment vector is fairly strong for all peptides, pointing toward the hydrophobic helix face.

The activity and selectivity of our nine peptides and the control MP1 peptide (gray points) was assessed under the same conditions on the PC-3 prostate cancer cell line (Figure 3). The same range of peptide concentrations was also tested on primary fibroblasts, to address the specificity of anticancer properties (Figure 4). Interestingly, MP1 appears to have a relatively low anticancer toxicity, similar to published data [82] on PC-3 cells. In contrast, all of our peptides had a much higher anticancer activity, with the most potent effect shown by ZYK1 and PEXA (Table 3). This toxic effect on cells was considerably lower for fibroblasts, and even lower on hematocytes than on PC-3 cancer cells (Table 2 and Table 3). The highest selectivity against cancer cells in absolute terms and relative to MP1 was achieved by T2R1 and ZYK1 (Table 3). Together with ZYK1 and PEXA, the A1A, FLEX, T2R1, and PG2 peptides also have preferential toxicity to cancer cells relative to human erythrocytes (HC20/IC20_C_). Only the KIA1 did not show any selectivity for prostate cancer cells (TI = 1). Non-homologous peptides showing the same or higher anticancer selectivity than the MP1 control (all but PG2, KIA1, and T2) can be examined in their helical wheel conformation for possible common features (Figure 5). Each of these six peptides has some advantageous performance aspect compared with the parent peptide, as discussed below.

## 4. Discussion

There are many published reports of anuran AMPs with anticancer activity and selectivity against PC-3 prostate cancer cells [85]. The present paper is the first report about good anticancer activity and selectivity of flexampin [22], a designed analog of adepantin-1 [26,53], and most importantly of novel AMPs found in the *Trichoplax adhaerens* proteome.

In view of drug development, the absence of significant toxicity is just as important as a good antibacterial activity or anticancer activity. The lack of specificity between prokaryotic and eukaryotic cells for many native AMPs opened the challenge to find still unknown native host defense peptides [48] (trichoplaxins from this work), and to design more selective analogs of native or artificial AMPs. We used the software tools previously developed by DJ to purposefully design antimicrobial peptides, with similar or better performance than pexiganan, the well-known peptide antibiotic close to use in approved clinical applications for topical treatments.

### 4.1. Effect of Charge and Helical Content on Activity

For all of the nine most potent of our new peptides, we detected strong anticancer activity against prostate cancer PC-3 cells. High cationic charge (from +6 for MAPA to +9 for PEXA) and high predicted amphipathic helix (percentages from 72% for T2R1 to 86% for ZYK1) for peptides that can span the membrane in helical conformation (from 18 residues for MAPA to 23 residues for A1A) are enough to ensure the anticancer activity, regardless of very different peptide sources or design procedures. The amphipathic helix conformation has been confirmed as the membrane-active conformation by other authors, and us for four peptides from our list of 10 peptides (see the last paragraph of Section 3.1). Except for trichoplaxins, remaining peptides were designed using our dedicated software tools with in-built restriction, to allow only for such substitutions that will increase amphipathic helicity of a parent peptide, known as to assume such a conformation when in close association with a membrane. Thus, despite an unorthodox SPLIT tool adoption to predict amphipathic peptides’ helical content, it is probably more accurate than the assumption of the 100% helical conformation for widely used calculations of the hydrophobic moment. Another common point for our nine peptides is consensus AMP, CPP, and anticancer peptide predictions, which, together with test results, suggest that interactions of cationic helical peptides and penetration into anionic membranes connect all these activities.

In contrast to our best peptides, the net charge of the Polybia-MP1 model anticancer peptide is only +2, and it has the weakest tendency to form an amphipathic α-helix. This might be one reason why the MP1 control peptide had the weakest anticancer activity among all of the tested peptides. When IC_50_ concentrations are compared, the nine peptides we tested have four (KIA1) to 40 times (ZYK1) stronger anticancer activity in vitro than the MP1 peptide. For the MP1 peptide, our measured IC_50_ = 60 µM is similar to the previously observed value of IC_50_ = 65 µM for growth inhibition [82]. It was argued that the presence and sequence location of two aspartates are the crucial features distinguishing MP1 from other mastoparans in low hemolicity [76], and in enhancing the peptide-peptide and peptide-membrane interactions for anionic membranes [86,87].

Differences in the therapeutic index (selectivity) are more challenging to understand and predict. Our results suggest the role of anionic residues (Glu or Asp) in increasing antimicrobial and anticancer selectivity for four out of six of the best peptides. Furthermore, less than perfect separation of helical polar and nonpolar faces can cause selectivity increase in the antimicrobial activity [88]. The “selectivity determinants” [89] are responsible for somewhat decreased amphipathicity and lesser membrane damage [90]. The same selectivity determinants can be considered as the candidate features for increasing the therapeutic index when activities are compared against normal and cancer cells.

### 4.2. Effect of Amphipathic Motifs on Activity

Can peptide conformation and activity/selectivity results help to identify the critical selectivity-determining residues? For T2R1 and PEXA, the selectivity seems to be connected to an unusually high number of arginines or lysines, that are not perfectly aligned at the helical polar face. For PEXA, leucine-18 is introduced in the middle of the polar sector, reducing the mean hydrophobic moment from 0.674 (for pexiganan) to 0.553 (HeliQuest results). Both terminal cationic residues of T2R1 are separated from the main group of polar face arginines. Arginine-1 addition to T2 decreases the hydrophobic moment from 0.436 (for T2) to 0.398 (HeliQuest results). Is this small change in amphipathicity responsible for the considerably higher selectivity of the T2R1 peptide? T2R1 is the best among the tested peptides for its selectivity against the PC-3 prostate cancer cell line. Something else, in addition to imperfect amphipathicity, controls the selectivity increase for certain arginine or lysine-rich peptides.

The cell-penetrating ability is inherent to some amphipathic peptides with a high percentage of cationic residues. More than five arginines are required to direct membrane penetration through the eukaryotic membrane [91]. This condition is fulfilled by guananin 2, the PenArg analog of penetratin, and T2R1, but not by T2. Penetratin, with the sequence RQIKIWFQNRRMKWKK, is a well-known cell-penetrating peptide [92], which is furthermore an antimicrobial [93,94] and anticancer peptide. That peptide is, to some degree, toxic to HeLa and Jurkat cancer cell lines [95]. The toxicity to HeLa cells increased almost 10 times after replacing all lysines with arginines, with a concomitant four-fold increase in the antimicrobial activity against *E. coli* and *S. aureus* of the PenArg analog [93]. Another arginine-rich peptide, guananin 2, has been recently designed as a promising candidate for drug development [96]. Its sequence, RQYMRQIEQALRYGYRISRR, also has the N-terminal arginine and a total of six to seven arginines, just like Pen Arg and T2R1. The guananin 2 mechanism of action involves a permeation of bacterial cytoplasmic membrane and low toxicity to human erythrocytes, resulting in a good selectivity index of about 24, better than the results that we obtained for PG2 and MAPA, and worse than our results with our other designed peptides. Interestingly, guananin 2 has arginine motifs R1-XXX-R5, R5-XXXXXX-R12, and R12-XXXXXX-R19, that are identical to T2R1 motifs R1-XXX-R5, R6-XXXXXX-R13, and R9-XXXXXX-R16. Arginine-zipper motifs serve to establish the intermolecular cation-π interactions [97], likely to be important for the interaction of anticancer and antibiotic peptides with membrane phospholipids.

The cationic twin motifs can also be the selectivity determinants. The RR and KK doublets appear 34 and 14 times, respectively, among 744 tumor-homing peptides [98]. In a more recently updated APD3 database of all natural antimicrobial peptides, the KK doublet appears 66 times among 230 anticancer AMPs. In most cases, it is bracketed with hydrophobic residues, which results in a high predicted alpha hydrophobic moment for a sequence segment containing the KK doublet. For A1A, PEXA, MAPA, T2R1, and ZYK1 (this work), and FLEX [22], one or more doublets of cationic residues combine with hydrophobic residues in such a way to facilitate predicted folding into an amphipathic helical conformation.

Future research might focus on the functional importance of common structural features, such as the presence of RR or KK doublets or cationic-zipper motifs of the type [K,R]-X_3_-[K,R] and [K,R]-X_6_-[K,R], in anticancer AMPs. Including more than a single arginine from the *Trichoplax adhaerence* sequence would help address whether some of these natural peptides with the polyarginine motif at their N-terminal can serve as peptide carriers for anti-cancer drugs [99]. It would also be useful to examine the serum stability of our best peptides before and after chemical modifications designed to increase their stability, as was done for MP1 [77,78].

### 4.3. Specific Advantages of Novel Folds

The MAPA analog of the MAP cell-penetrating peptide is five times less hemolytic and has at least two times stronger antibacterial activity than MAP [73] (Table 2). Accordingly, by introduced substitutions, we increased the selectivity index by at least one order of magnitude, and converted a strongly hemolytic, predominantly cell-penetrating peptide with no antibacterial selectivity (SI = 1, [73]), into a potent peptide antibiotic. MAP is an important molecule for engineering new anticancer agents [72,100], but as far as we can tell, MAPA is the first known selective anticancer MAP analog. It remains to be seen if MAPA can still be used as a vector to deliver drugs to intracellular targets, with possible additional multiplication of therapeutic index for a conjugated peptide.

After being designed as a broad-spectrum peptide antibiotic [55], Koszalka et al. reported, in 2011, that pexiganan also functions as an anticancer peptide [101]. Pexiganan’s IC_50_ = 4 μM against lymphoma cell line U937 is identical to the IC_50_ that we observed for the PEXA analog acting against prostate cancer PC-3 cells. However, Koszalka and coauthors (2011) did not compare the survival of healthy human fibroblasts and U937 cells to determine the therapeutic index. We obtained a therapeutic index of 3 for PEXA. The PEXA structure was published in 2011 as a promising AMP [23], but PEXA was first synthesized and tested in this work for its antibiotic and anticancer activity and selectivity.

In the ZYK1 example, the parent peptide already has selective antimicrobial and antitumor activity [102]. The IC_50_ ranges from 15 to 25 μM against two selected cancer cell lines: HeLa (human cervical carcinoma cells) and HL60 (human promyelocytic leukemia cells). Minimal cytotoxicity was observed for model normal host cells (NIH 3T3 cell line), but without data points that would allow the therapeutic index calculation. In a recent paper by these authors, the reported cytotoxicity to human primary cells (human neonate chondrocytes) was IC_50_ > 40 μM [59]. It follows that the TI for the parent peptide is somewhat higher than 2. MIC values were close to 10 μM for Gram-negative and Gram-positive bacteria [103]. After using putative bacteriocin to extend the helical length of the parent peptide, the ZYK1 peptide had about 10 times stronger anticancer activity and several times higher therapeutic index, that parallels about 10 times stronger broad-spectrum antimicrobial activity. However, ZYK1 had no apparent distinction in the antibacterial tests.

Higher anticancer activity and selectivity of FLEX with respect to A1A peptide parallels the higher FLEX antimicrobial activity and selectivity. Possible reasons have been discussed above for the achieved higher therapeutic index of T2R1, after the addition of the N-terminal arginine to T2. If our highly cationic peptides are, to some degree, cell-penetrating peptides, they are likely to have an easier task to reach the mitochondria of cancer cells, because of an enhanced net negative surface charge in cancer cells [8]. The inhibition of bioenergetics [104] can bring together the mechanism of action for some antibiotic and anticancer peptides.

## 5. Conclusions

The peptides presented in this work offer a wide spectrum of novel helical structures as lead compounds for fine-tuning their anticancer potential. Screening for therapeutic index and overall performance singled out T2R1 and ZYK1, respectively, as the best anticancer peptides. In future research, closer scrutiny can be devoted to the deep evolutionary origin of templates used for constructing these peptides.

In conclusion, all nine potent antimicrobial peptides that we selected for this work have high toxicity to cancer cells. The therapeutic index for cancer cells is not as high as the selectivity index for bacteria. Nevertheless, with the exception of the two kiadins (PG2 and KIA1), it is equal or higher than the TI for the anticancer control peptide MP1. On top of their good selectivity, they also had much higher toxicity towards cancer cells than MP1, which indicates that they could be used at a lower concentration than other anticancer peptides such as MP1. Hence, it is a promising approach to consider bacterial membranes as functional analogs of cancer cell membranes [105]. Overall, it appears, from this study, that both these peptides and the method that allowed their design carry significant potential for medical use, as new types of antibiotics and as selective anticancer drugs.

## Figures and Tables

**Table 1:** Name, sequence, origin, amphiphatic helix prediction, prediction of antimicrobial, anticancer, and cell-penetrating activity for nine designed antimicrobial peptides and one known anticancer peptide.

Name [Abbreviation]	A Sequence with Added or Substituted Residues in Bold Font and Underlined	Reference *	Amphipathic Helix Pred. **	SMIC ^¶^ Template/Peptide	Anticancer Prediction ^&^	CAMP_R3_ AMP Pred. All AI Classifiers ^$^	CPP Prediction ^#^
Trichoplaxin-2 [T2]	HHWRRYARIGFRAVRTVIGK-NH_2_	This work	75%	138	0.65/0.98/0.96	>0.85 Yes	0.73/0.88
Trichoplaxin-2A [T2R1]	**R**HHWRRYARIGFRAVRTVIGK-NH_2_	This work	72%	138/58	0.85/0.98/0.96	>0.87 Yes	0.73/0.91
Adepantin-1A [A1A]	GI**K**K**A**VGKALKGLKGLLK**A**LGES-NH_2_	This work	78%	513/517	0.24/0.98/1.0	>0.91 Yes	0.93/0.39
Pexiganan-L18 [PEXA]	GIGKFLKKAKKFGKAFV**L**ILKK-NH_2_	[23]	77%	352/435	0.82/0.98/1.0	>0.99 Yes	0.91/0.75
Flexampin [FLEX]	GI**KKWV**KGVAKGVAKDLA**K**K**I**L-NH_2_	[22]	82%	170/1460	0.29/0.98/1.0	≥0.99 Yes	0.74/0.56
Zyk-1 [ZYK1]	**GIGRE**IIKKIIKKI**G**KKI**GRII**-NH_2_	This work	86%	180/1098	0.43/0.97/0.99	>0.97 Yes	0.83/0.64
*Di*PGLa-H [PG2]	KIAKVALKAL**KIAKVALKAL**-NH_2_	[24]	75%	16/204	0.61/0.98/0.99	>0.48	0.94/0.85
Kiadin-1 [KIA1]	KIAKVALKAL**KIAKGALKAL**-NH_2_	[24]	80%	16/251	0.61/0.98/0.99	>0.48	0.97/0.86
Mapegin [MAPA]	K**IGK**K**I**LKALK**G**ALK**E**LA-NH_2_	This work	78%	92/253	0.74/0.98/1.0	>0.59 Yes	0.95/0.71
Polybia-MP1 [MP1]	IDWKKLLDAAKQIL-NH_2_	[45]	50%	25	0.91/0.98/0.95	>0.78 Yes	0.57/0.53

* The pexiganan-L18, flexampin, DiPGLa-H, and kiadin-1 sequences were published before, along with corresponding antimicrobial activity and selectivity tests (testing was not performed before for the pexiganan analog) [22,23]. Known anticancer peptide polybia-MP1 [45] was used as the control. ** Sequence percentage predicted to fold into an amphipathic helical conformation is determined from the cut-off of 2.0 for the INDA sequence profile of modified hydrophobic moment obtained by the SPLIT 3.5 predictor. ^¶^ SMIC composite index: ((Max INDA) × (number of >2.0 INDA residues) from SPLIT 3.5) × (SI from MUTATOR)**/**(MIC from MIC-predictor with peptide extended with CKITGC). ^&^ Anticancer prediction probabilities are separated by slash symbols from left to right according to servers provided by [34] Tyagi et al. (2013), [35] Schaduangrat et al. (2012), and [36] Boopathi et al. (2019). ^$^ AMP prediction according to CAMP_R3_ four artificial intelligence algorithms [31] with “Yes” annotation when all four servers predict the AMP class or AMP probability greater than 0.5. ^#^ CPP prediction according to CPPrex-FL [31] (or SkipCPP-Pred) (first number) and MLCPP [33] algorithms (second number). The only non-CPP prediction is in a gray shade.

**Table 2:** MIC and hemolicity HC values (in µM) are presented, together with the selectivity SI, and selectivity-activity products (the performance PE) for peptides (see Table 1 for abbreviations) tested on standard bacterial strains, multidrug-resistant clinical isolates, and human erythrocytes.

	T2	T2R1	A1A	PEXA	FLEX	ZYK1	PG2	KIA1	MAPA	MP1
**MIC (*E. coli ATCC 25922*)**	0.5–1	1	1	0.5–1	0.25	1	1.5	0.75	1–2	
**MIC (*E. coliMG1655*)**	>32	4	2	4	16	4	16	8	4	>32
**MIC (*E. coli clin. isolate*)**	8	4	32	4	0.5	2	6	12	8–16	
**MIC (*P. aerug. ATCC 27853*)**	4	1	64	4	2	16	6	6	32–64	
**MIC (*P. aerug. clin. isolate*)**	32	8	>64	16	2–4	16	6	3	32	
**MIC (*K. pneum. ATCC 13883*)**	4	2	4	2	0.5–1	2	3	3	8	
**MIC (*K. pneum. clin. isolate*)**	8	2–4	8	4	2–4	4	12	12	8	
**MIC (*A. baum. ATCC 19606*)**	1	2	2	1–2	0.5–1	2	1.5	1.5	1–2	
**MIC (*A. baum. clin. isolate*)**	8	8	4–8	1–2	1	2	1.5–3	1.5	4	
**MIC (*S. aureus ATCC 29213*)**	1	0.5	1	0.5	0.25	1	0.75	1	0.5	
**MIC (*S. aureus clin. isolate*)**	4	4	4	2	4	2	1.5	3	8	
**HC10**	1.4	3	25	1.6	6	3	3	3	1.7	20
**HC20**	3	25	80	7	25	8	14	6	3	37
HC50 *	28	7000 *	125 *	520 *	1600 *	29	18	20	20	170
**SI_c_ = HC20/MIC(coli) ^&^**	4	25	80	9.3	100	8	9.3	8	2	
**SI_a_ = HC20/MIC(aureus) ^&^**	3	50	80	14	100	8	18.7	6	6	
**PE_c_(20) = SI_c_/MIC(coli) ^$^**	5.3	25	80	12.4	400	8	3.1	10.7	1.3	
**PE_a_(20) = SI/MIC(aureus) ^$^**	3	100	80	24.9	400	8	24.9	6	12	

* HC50 values are extrapolated if all concentrations tested lysed less than 50% of cells. ^&^ Selectivity index SI (20) values are calculated as the ratio of HC_20_ peptide concentration (that is causing 20% hemolysis) to the minimal inhibitory concentration (MIC) for *E. coli* ATCC 25922 (SI_c_), or for *S. aureus* ATCC 29213 (SI_a_). ^$^ Overall peptide performance PE (20) values (expressed in μM^−1^) are calculated as the product of selectivity SI (20) and antibacterial activity 1/MIC.

**Table 3:** IC50 (µM) and therapeutic index (TI) of designed peptides, when activities are compared against human fibroblasts (1/IC50_F_) and human PC-3 cancer cells (1/IC50_C_). We defined the performance as the product of TI and anticancer activity (1/IC50_C_).

	T2	T2R1	A1A	PEXA	FLEX	ZYK1	PG2	KIA1	MAPA	MP1
**IC50_F_ (Fibroblasts)**	35	80	30	12	30	10	10	15	25	150
**IC50_C_ (PC-3)**	10	8	12	4	6.25	1.5	6	15	8	60
**TI (IC50_F_/IC50_C_)**	3.5	10	2.5	3	4.8	6.7	1.7	1.0	3.1	2.5
**TI (HC20/IC20_C_)**	1	5	27	6.7	25	8.6	6	1	1	1
**TI/ IC50_C_**	0.35	1.25	0.21	0.75	0.77	4.47	0.28	0.07	0.39	0.04
(TI/ IC50_C_) vs. MP1 *	8.4	30	5	18	18.5	107	6.8	1.6	9.3	1.0

* Relative performance with respect to MP1 control peptide.
